# Supplementary material for: Inhibitory Neural Network’s Impairments at Hippocampal CA1 LTP in an Aged Transgenic Mouse Model of Alzheimer’s Disease
Source: Int J Mol Sci. 2021 Jan 12;22(2):698. doi: 10.3390/ijms22020698 (PMC7828160; doi:10.3390/ijms22020698)
Supplement: Supplementary file 1 [file ijms-22-00698-s001.pdf]

International Journal of *Molecular Sciences*

MDPI

Supplementary Materials

Inhibitory Neural Network's Impairments at Hippocampal CA1 LTP in an Aged Transgenic Mouse Model of Alzheimer's Disease

Hyeon Jeong Seo [1,†], Jung Eun Park [2,3,†], Seong-Min Choi [4,5], Taekyoung Kim [5], Soo Hyun Cho [4,5], Kyung-Hwa Lee [6], Woo Keun Song [7], Juhyun Song [8], Han-Seong Jeong [9], Dong Hyun Kim [10,*],and Byeong C. Kim [1,4,5,*]

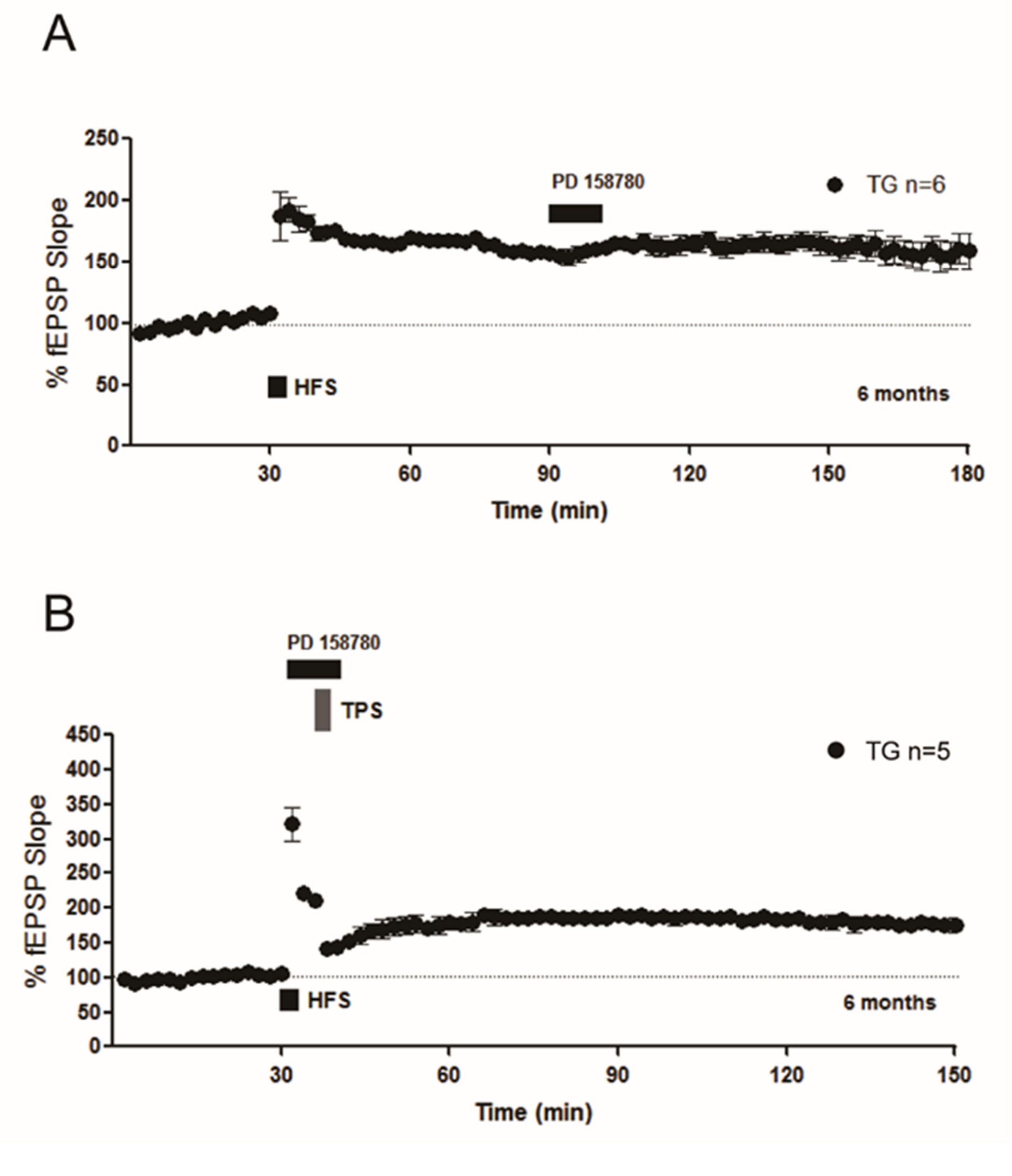

Figure S1. Effects of depotentiation on NRG1-ErbB signaling. (**A**) After 1 h LTP induction, 10 μM PD158780 was perfused and observed for 2 h (*n* = 6). (**B**) TPS-induction was measured with 10 min PD158780 perfusion starting 2 min prior to HFS application (*n* = 5).

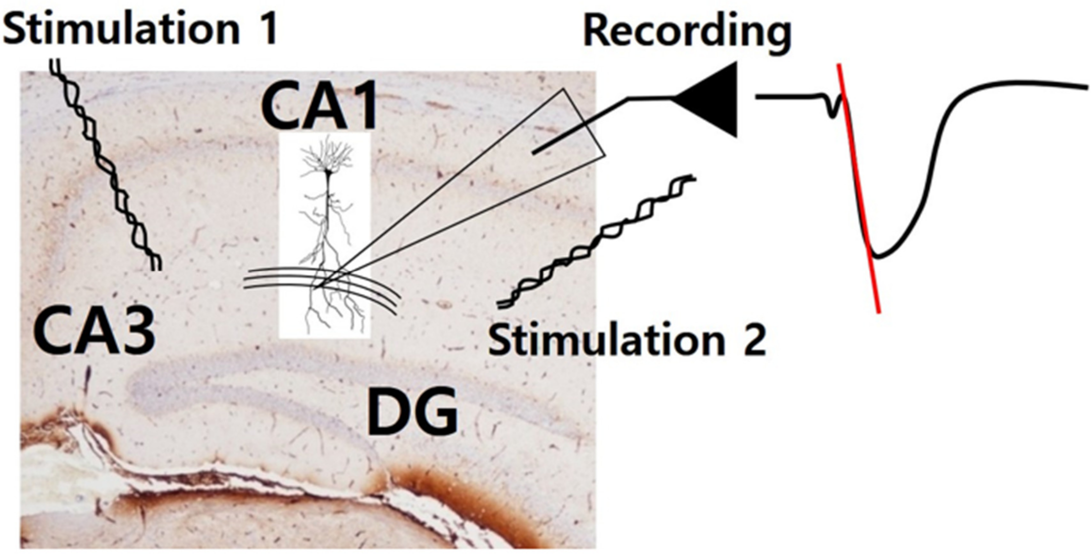

Figure S2. Schematic image of stimulating and recording electrodes. The field excitatory postsynaptic potentials (fEPSPs) were recorded with extracellular electrodes using mice hippocampal slices. The stimulating electrodes were placed in the Schaffer collateral pathway (Stimulation 1) and in the subiculum area (Stimulation 2). The recording electrode was placed in the CA1 pyramidal cells, and recorded the slope of fEPSPs.
